# Global Patterns and Predictions of Seafloor Biomass Using Random Forests

**DOI:** 10.1371/journal.pone.0015323

**Published:** 2010-12-30

**Authors:** Chih-Lin Wei, Gilbert T. Rowe, Elva Escobar-Briones, Antje Boetius, Thomas Soltwedel, M. Julian Caley, Yousria Soliman, Falk Huettmann, Fangyuan Qu, Zishan Yu, C. Roland Pitcher, Richard L. Haedrich, Mary K. Wicksten, Michael A. Rex, Jeffrey G. Baguley, Jyotsna Sharma, Roberto Danovaro, Ian R. MacDonald, Clifton C. Nunnally, Jody W. Deming, Paul Montagna, Mélanie Lévesque, Jan Marcin Weslawski, Maria Wlodarska-Kowalczuk, Baban S. Ingole, Brian J. Bett, David S. M. Billett, Andrew Yool, Bodil A. Bluhm, Katrin Iken, Bhavani E. Narayanaswamy

**Affiliations:** 1 Department of Oceanography, Texas A&M University, College Station, Texas, United States of America; 2 Department of Marine Biology, Texas A&M University at Galveston, Galveston, Texas, United States of America; 3 Instituto de Ciencias del Mar y Limnología, Universidad Nacional Autónoma de México, México D.F., México; 4 Alfred Wegener Institute for Polar and Marine Research, Bremerhaven, Germany; 5 Australian Institute of Marine Science, Townsville, Queensland, Australia; 6 Biological and Environmental Sciences, Qatar University, Doha, Qatar; 7 Biology and Wildlife Department, Institute of Arctic Biology, University of Alaska Fairbanks, Fairbanks, Alaska, United States of America; 8 College of Marine Life Science, Ocean University of Qingdao, Qingdao, China; 9 CSIRO Marine and Atmospheric Research, Cleveland, Queensland, Australia; 10 Department of Biology, Memorial University, St. John's, Newfoundland and Labrador, Canada; 11 Department of Biology, Texas A&M University, College Station, Texas, United States of America; 12 Department of Biology, University of Massachusetts, Boston, Massachusetts, United States of America; 13 Department of Biology, University of Nevada Reno, Reno, Nevada, United States of America; 14 Department of Biology, University of Texas at San Antonio, San Antonio, Texas, United States of America; 15 Department of Marine Sciences, Polytechnic University of Marche, Ancona, Italy; 16 Department of Oceanography, Florida State University, Tallahassee, Florida, United States of America; 17 Department of Oceanography, University of Washington, Seattle, Washington, United States of America; 18 Harte Research Institute, Texas A&M University-Corpus Christi, Corpus Christi, Texas, United States of America; 19 Institut des sciences de la mer de Rimouski, Université du Québec à Rimouski, Rimouski, Québec, Canada; 20 Institute of Oceanology, Polish Academy of Sciences, Sopot, Poland; 21 National Institute of Oceanography, Dona Paula, Goa, India; 22 National Oceanography Centre, Southampton, Southampton, United Kingdom; 23 School of Fisheries and Ocean Sciences, University of Alaska Fairbanks, Fairbanks, Alaska, United States of America; 24 Scottish Association for Marine Science, Scottish Marine Institute, Oban, United Kingdom; Dalhousie University, Canada

## Abstract

A comprehensive seafloor biomass and abundance database has been constructed from 24 oceanographic institutions worldwide within the Census of Marine Life (CoML) field projects. The machine-learning algorithm, Random Forests, was employed to model and predict seafloor standing stocks from surface primary production, water-column integrated and export particulate organic matter (POM), seafloor relief, and bottom water properties. The predictive models explain 63% to 88% of stock variance among the major size groups. Individual and composite maps of predicted global seafloor biomass and abundance are generated for bacteria, meiofauna, macrofauna, and megafauna (invertebrates and fishes). Patterns of benthic standing stocks were positive functions of surface primary production and delivery of the particulate organic carbon (POC) flux to the seafloor. At a regional scale, the census maps illustrate that integrated biomass is highest at the poles, on continental margins associated with coastal upwelling and with broad zones associated with equatorial divergence. Lowest values are consistently encountered on the central abyssal plains of major ocean basins The shift of biomass dominance groups with depth is shown to be affected by the decrease in average body size rather than abundance, presumably due to decrease in quantity and quality of food supply. This biomass census and associated maps are vital components of mechanistic deep-sea food web models and global carbon cycling, and as such provide fundamental information that can be incorporated into evidence-based management.

## Introduction

### Rationale

A ‘census’, according to our dictionaries, was originally a counting of individuals for the purpose of taxation. The Census of Marine Life (CoML) is on the other hand an attempt to make a comprehensive assessment of what lives in the world's oceans. CoML is attempting to document, describe, list, archive and map as many species of organisms as possible in all marine ecosystems, independent of an individual species' population size. A natural by-product of CoML however has been new tabulations of animal abundances and biomass by CoML field projects. The purpose of this CoML biomass synthesis has been to capture all the new information on biomass that has been uncovered during CoML into a single data base, independent of species composition. This project has thus archived and mapped a broad spectrum of biomass data from CoML projects from around the world, added data from a number of previous comprehensive reviews, and, as a result, produced maps of biomass of a limited number of size groups living on the sea floor on a world wide basis.

While the causes of biodiversity remain obscure to a large degree, there is general agreement that biomass is a function of food supply to or within any particular habitat. As a result, standing stock biomass has been used as a surrogate for biomass production and carbon flow to and through an ecosystem, without necessarily defining the taxa contributing to the biomass. On the other hand, by analyzing the statistical relationships of diversity to biomass, it might be possible to make some practical inferences about the effects that productivity might have on diversity [1], as this is an open question that has generated considerable conjecture [2]. While the biomass census is not related to ‘taxation’ in the classic sense, it directly links marine populations to carbon as an ecosystem model currency. Inorganic carbon is fixed into organic-rich compounds by photosynthesis and then transferred through food webs where it has a variety of fates, usually a return to CO_2_. However, it is also harvested by fishers and it thus ends up in markets around the world. A biomass census therefore has relevance to societies because human populations are putting a ‘tax’ on the ocean biota in the form of valuable protein in fisheries products.

### Historical background

The earliest quantitative sampling of the sea floor began at the beginning of the 20^th^ century as an attempt to determine food resources available to bottom-dwelling fish in European waters [3], [4]. A good review of the mechanical instruments developed for the early shallow-water surveys [5] pictures a wide variety of grab-like samplers, many still in use today. By the middle of the 20^th^ century, the macrofauna of many continental shelves and estuaries had been sampled quantitatively by a relatively standard set of instruments. For demersal fishes and vagile megafaunal invertebrates, the most common sampling methods are trawling and photography. Both methods have weaknesses: for example, trawling tends to capture only surface-dwelling and slow species. It may be impossible to positively identify animals to species from photographs. However, to this day neither is fool proof. With smaller forms (meiofauna, microfauna, bacteria and viruses), sampling problems are solved seemingly easily by utilizing small-diameter cores, but care has to be taken not to lose organisms by either washing or bow-wake of sampling devices. For these groups, the problem is that they have not yet been sampled comprehensively on global or ocean-basin scales.

Generalizations about the controls of sea floor biomass began to emerge by the middle of the 20^th^ century. Expeditions sponsored by Union of Soviet Socialist Republics (USSR: dissolved in 1991) reached every corner of the globe. This large body of work concluded that biomass declines sharply with depth and with distance from land. They observed that high latitudes tended to have higher biomass than low latitudes. The major food supply to both pelagic and sea floor communities was the rain of particulate detritus, enhanced by a ladder of vertical migration [6]. Sea floor biomass likewise declines precipitously with depth, but is also tightly coupled to primary production in surface layers. Regression equations of the variation in benthic biomass as a function of depth and primary production established in the 1970's initially (reviewed in [7]) are still reasonable estimates of deep benthic biomass today [8]. The slopes of the biomass regressions have been equated to the rate at which the delivery of POC to the sea floor declines, but the height or zero intercept of the regression line is a function of the mean primary production in the photic zone.

Previous reviews of seafloor standing stocks focused on bathymetric standing stock patterns in which the distribution of biomass and abundance was fitted to a linear function of water depth or direct measurement of sinking particle flux [7], [8], [9], [10]. Applying such equations is conceptually intuitive but the relationships tend sometimes to fall apart in large scale predictive mapping. In this paper, we explore a novel machine-learning algorithm, Random Forests [11], to model the complex and potentially non-linear relationships between oceanic properties and seafloor standing stocks. Random Forests (RF) is a data mining method widely used in the fields of bioinformatics [12], speech recognition [13], and drug design and development [14]. Recently RF is gaining popularity in terrestrial ecology [15], [16], [17]; however, so far, only a handful of studies have applied RF in marine ecosystems [18], [19]. In short, RF, as the name suggested, is an ensemble of many decision trees with binary divisions. Each tree is grown from a bootstrap sample of response variable and each node is guided by a predictor value to maximize differences in offspring branches. The fit of the tree is examined using the data not in the bootstrap selection; hence, cross-validation with external data is not necessary. Predictive accuracy requires low bias and low correlation between decision trees [11]. RF achieves these by growing a large number of trees and then averaging the predictions. At the same time, the node decision is chosen from a random subset of predictors to make the trees look as different as possible. RF does not assume any data distribution and does not require formal selection of predictors. RF is robust to outlier and unbalanced data, making it a better choice than traditional statistical methods [12].

## Materials and Methods

### Response Variables

**Figure 1:**
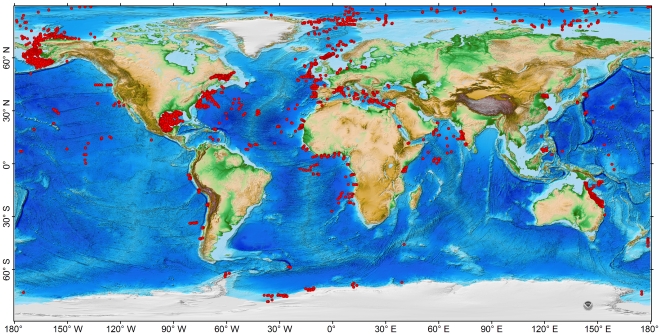
Distribution of abundance and biomass records in the “CoML Fresh Biomass Database”. References and locations for each size class are given in Appendix S1 and File S1. Bathymetric layer uses NOAA ETOPO 1 Global Relief Model [26].

Biomass and abundance of bacteria, meiofauna, macrofauna, megafauna (invertebrates+fishes), invertebrates, and fishes were assembled from literature and the Census of Marine Life (CoML) field projects (Figure 1 and Appendix S1). The “CoML Fresh Biomass Database” includes 4872 biomass records, 5511 abundance records, and 4196 records with both biomass and abundance from 175 studies. Additional datasets include nematodes (230 records from 10 studies) and pelagic decapods (17 records from 1 study); however, they were not included in this analysis. The complete list of references and detailed data information are available in Appendix S1 and File S1.

Categories of benthic fauna are usually defined by size classes. In this paper, we refer to the term “bacteria” to include both bacterial and archaeal domains. We have not included viruses. The metazoan meiofauna and macrofauna are small infauna invertebrates sampled by core or grab devices and retained on 20 to 74-µm and 250 to 520-µm sieves, respectively. Megafauna refers to large epibenthic invertebrates and demersal fishes (usually larger than 1 cm) caught or recorded by bottom trawling and photographic survey. Many studies deal with trawl invertebrates and fishes separately; hence, 3 categories were created for the megafauna, including the invertebrates plus fishes, invertebrates, and fishes. Here the “megafauna” dataset includes both invertebrates and fishes. Estimates of meiofaunal and macrofaunal standing stocks are affected by the gear design, sampling area, and sieve sizes [7], [20], [21], [22]. These factors however have been suggested to be minor compared to water depth at a global scale and do not significantly affect the overall level and pattern of stock-depth relationships [2], [8]. Only studies reporting standing stocks for the whole assemblage of a size category were used in these analyses. Benthic foraminiferans were not included due to difficulty differentiating between living biomass from empty tests or shells [8], [9]. Throughout this analysis, the abundance was standardized to cells (for bacteria) or individuals (for meiofauna, macrofauna, and megafauna) per square meter. The biomass was standardized to milligrams carbon per square meter using appropriate conversion factors from wet or dry weight to organic carbon weight [7], [9].

### Environmental Predictors

Environmental variables with global coverage were utilized to characterize 1) the surface ocean climate relating to phytoplankton production, 2) water column processes associated with export POC flux, 3) bottom water properties characterizing the seafloor habitats, and 4) seafloor relief (water depth) as a proxy of declining export POC flux arriving at the ocean floor (Table 1 and Figure S1). Contemporaneous environmental and standing stocks data were not always available; therefore, mean and standard deviation (S.D.) of the predictors were calculated for the longest time periods possible. The variables are listed as:

1. Primary productivity variables: Decadal mean and standard deviation (S.D.) of monthly net primary production (NPP) models (cbpm, vgpm), and the data inputs for the NPP models [23], [24] including chlorophyll concentration (chl), sea surface temperature (sst), photosynthetic available irradiance (par), mixed layer depth (mld), particle backscatter (bbp), phytoplankton growth rate (growth), and carbon concentration (carbon), all calculated between years of 1998 and 2007. The monthly data were obtained from the Ocean Productivity Group, Oregon State University, as products of the Sea Viewing Wide Field-of-view Sensor (SeaWiFS r2009.1) and Advanced Very High Resolution Radiometer (AVHRR).
2. Water column processes: Decadal mean of water-column integrated total carbon (int.c) and nitrogen (int.n), detrital carbon (det.c) and nitrogen (det.n), phytoplankton (phyt) and zooplankton (zoop), as well as export flux of detrital carbon (det.flx.c) and nitrogen (det.flx.n), obtained from a 10-year simulation of monthly model outputs from 1995 to 2004 using Ocean Circulation and Climate Advanced Model (OCCAM) driven by a nitrogen based Nutrient Phytoplankton Zooplankton Detritus (NPZD) Model [25].
3. Bottom water properties: Annual mean and seasonal standard deviation (S.D.) of bottom water temperature, salinity, oxygen, nitrate, phosphate, and silicate concentration were obtained from World Ocean Atlas 2009, NOAA National Oceanographic Data Center.
4. Global ocean depths were obtained from the ETOPO1 Global Relief Model, NOAA National Geophysical Data Center [26].

**Table 1:** Global datasets of environmental predictors.

Data Type	Data Source	Res.	Cell	Abbrev.	Variable	Unit
**Primary Production**	Ocean Productivity, OSU	5 minutes	3×3	chl	Chlorophyll a concentration (SeaWiFS r2009.1)	mg m^−3^
Decadal mean & standard deviation of monthly data from January 1998 to December 2007		5.3 minutes	3×3	sst	Sea Surface Temperature (AVHRR)	°C
		5 minutes	3×3	par	Photosynthetically available radiation (SeaWiFS r2009.1)	Einstein m^−2^ day^−1^
		5 minutes	3×3	bbp	Particulate backscatter (SeaWiFS r2009.1)	m^−1^
		10 minutes	1×1	mld	Mixed layed depth	m
		5 minutes	3×3	growth	Phytoplankton growth rate	divisions day^−1^
		5 minutes	3×3	carbon	Carbon concentration	mg m^−3^
		5 minutes	3×3	vgpm	Chlorophyll based net primary production	mg C m^−2^ day^−1^
		5 minutes	3×3	cbpm	Carbon based net primary production	mg C m^−2^ day^−1^
**Water column**	Yool et al. [25]	1 degree	1×1	int.c	Integrate C to 500 m above seafloor	mg C m^−2^
Decadal mean of monthly model simulation from January 1995 to December 2004		1 degree	1×1	int.n	Integrate N to 500 m above seafloor	mg N m^−2^
		1 degree	1×1	det.c	Integrate detrital C to 500 m above seafloor	mg C m^−2^
		1 degree	1×1	det.n	Integrate detrital N to 500 m above seafloor	mg N m^−2^
		1 degree	1×1	phyt	Integrate phytoplankton to 500 m above seafloor	mg N m^−2^
		1 degree	1×1	zoop	Integrate zooplankton to 500 m above seafloor	mg N m^−2^
		1 degree	1×1	det.c.flx	Detrital C flux at 500 m above seafloor	mg C m^−2^ day^−1^
		1 degree	1×1	det.n.flx	Detrital N flux at 500 m above seafloor	mg N m^−2^ day^−1^
**Bottom Water**	World Ocean Atlas 2009	1 degree	1×1	temp	Temperature	°C
Annual mean & seasonal standard deviation		1 degree	1×1	salin	Salinity	ppm
		1 degree	1×1	oxyg	Oxygen concentration	milliters liter^−1^
		1 degree	1×1	nitra	Nitrate concentration	micromoles liter^−1^
		1 degree	1×1	phos	Phosphate concentration	micromoles liter^−1^
		1 degree	1×1	si	Silicate concentration	micromoles liter^−1^
**Water Depth**	ETOPO1 Global Relief	1 minute	N.A.	depth	Water depth	m

The mean value was extracted for abundance and biomass records with catchment area of 3×3 or 1×1 cells. The datasets are divided into 4 categories, including 1) primary productivity variables, 2) water column variables, 3) bottom water properties, and 4) water depth. The table abbreviations follow: Res.  =  data resolution, Cell  =  cell size for extraction, Abbrev.  =  variable abbreviation.

### Data Analyses and Modeling

We used partial regression analysis to examine the relationships between standing stocks and depth when the latitude and longitude are held constant. The multiple regression residuals of stocks against latitude and longitude were used as dependent variables to regress against depth. To bring the dependent variable back to an appropriate scale, the y-intercept from the multiple regression was added to the residuals. The partial regression was also used in the pre-treatment of the depth-integrated bacteria data to standardize sediment penetration depths (from 0.5 to 29.5 cm; >83% are between 5 and 15 cm). Similar approaches has been developed and tested in Rex et al. [8].

A stochastic model between standing stocks and 39 environmental predictors (Table 1 and Figure S1) was constructed using Random Forests (RF) [11]. RF is a member of Regression Tree Analyses (RTA) [27]. In RTA, the response variable (standing stocks) is recursively partitioned into small successive binary splits. Each split is based on a single value of predictor from an exhaustive search of all available predictors to maximize the differences between the offspring branches. In RF, the response variable was bootstrap resampled before conducting RTA to generate large numbers of un-pruned decision trees (1000 trees in this study). Unlike traditional RTA, the RF algorithm searches the best split from a random subset of predictors (1/3 of all variables) and the prediction can be made from new data (environmental) by averaging the model outputs of all trees. At each bootstrap resampling step, 2/3 of the data (in-bag) were selected to build the decision tree. The other 1/3 of the data (out-of-bag, or OOB) were used to carry out an internal examination of model (decision tree) prediction error and estimate variable importance. The OOB data can generate predictions using the tree grown from the in-bag data. These OOB predictions were aggregated (by averaging the outputs of all trees) to compare with the observations and estimated the prediction error. The performance of the RF model was examined as percent variance explained: R^2^ = 1– MSE_OOB_/observed variance, where MSE_OOB_ is the mean square error between observations and OOB predictions. Predictor Importance was determined by how much worse the OOB predictions can be if the data for that predictor are randomly permuted. This essentially mimicked what would happen with or without the help of that predictor. The increase of prediction error (MSE_OOB_) after the permutation was used to measure its contribution to the prediction accuracy. This accuracy importance measure (increase of MSE_OOB_) was computed for each tree and averaged over the forest (1000 trees).

### Construction of Random Forest Models

Standing stocks (biomass and abundance) were logarithm (base 10) transformed before conducting RF analysis. Environmental data were extracted based on the latitude and longitude of the stock records by averaging a box of size 3×3 or 1×1 cells (Table 1). Mean value of the box was matched to the corresponding stocks record. RF algorithm was then run independently on each of the 12 datasets. Most primary productivity predictors have declining temporal coverage at the high latitudes between years of 1998 and 2007 due to prolonged winter darkness or cloud cover preventing SeaWiFS ocean color measurements (Figure S2). This can be a source of error during the RF modeling, because decadal mean and standard deviation of the predictors was only calculated from the available monthly data. In order to evaluate the model stability, we conducted 4 RF simulations for each dataset. The simulations were based on different data selection scenarios, including: 1) all standing stocks and environmental data were included; 2) only data calculated from >30 months of SeaWiFS measurements were included; 3) only data calculated from >60 months of SeaWiFS measurements were included; 4) only data calculated from >90 months of SeaWiFS measurements were included. In other words, Scenario 1 retained all the data and Scenario 4 excluded much of the high latitude data (>50°N or S, see Figure S2). The mean and standard deviation (S.D.) of the model performance (R^2^) and variable importance were calculated to evaluate the model sensitivity. In the following text, the “simulations” refer to the RF runs under the 4 data selection scenarios.

### Global Prediction of Seafloor Standing Stocks

Environmental data were averaged to the same grid resolution (1 arc degree grids) before using them as model inputs for global standing stocks predictions (Figure S1). For each dataset, 4 global predictions were generated from RF simulations. The mean and coefficient of variation (S.D./mean * 100%) were calculated for each grid to optimize the predictions and examine the output stability. In order to produce a smooth predicted surface, the predictions were interpolated to 0.1 degree cell resolution using Inverse Distance Weighting (IDW). The predicted map of standing stocks is displayed in color classes using Jenks Natural Breaks Optimization method to maximize the differences between the classes. The global integral of benthic biomass was integrated from each cell value multipling the cell area on predicted map based on equidistant cylindrical projection. The calculations were based on the formula: Global integral  = Σ map cell value (in per unit area) * cell area at equator (∼12343 km^2^) * cosine (latitude). Statistical analyses and RF modeling used R 2.11.0 [28] and R package randomForest [29]. Geostatistical analyses and mapping used ESRI® ArcMap™ 9.2 and R package sp [30].

## Results

### Partial linear regressions

**Figure 2:**
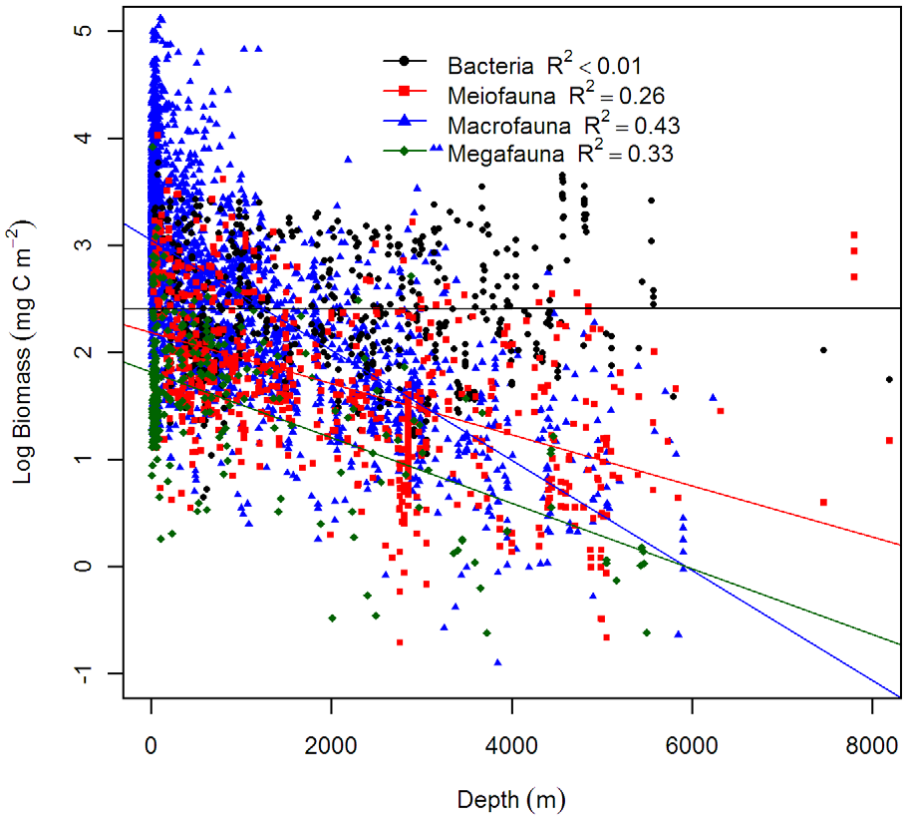
Biomass as a function of depth for bacteria, meiofauna, macrofauna, and megafauna. Biomass was log_10_ transformed and the effects of latitude and longitude were removed by partial regression. Figure legend follows Rex et al. [8] for comparison. References of data source are available in Appendix S1 and File S1. Regression equations and test statistics for each size categories are available in Table 2.

**Figure 3:**
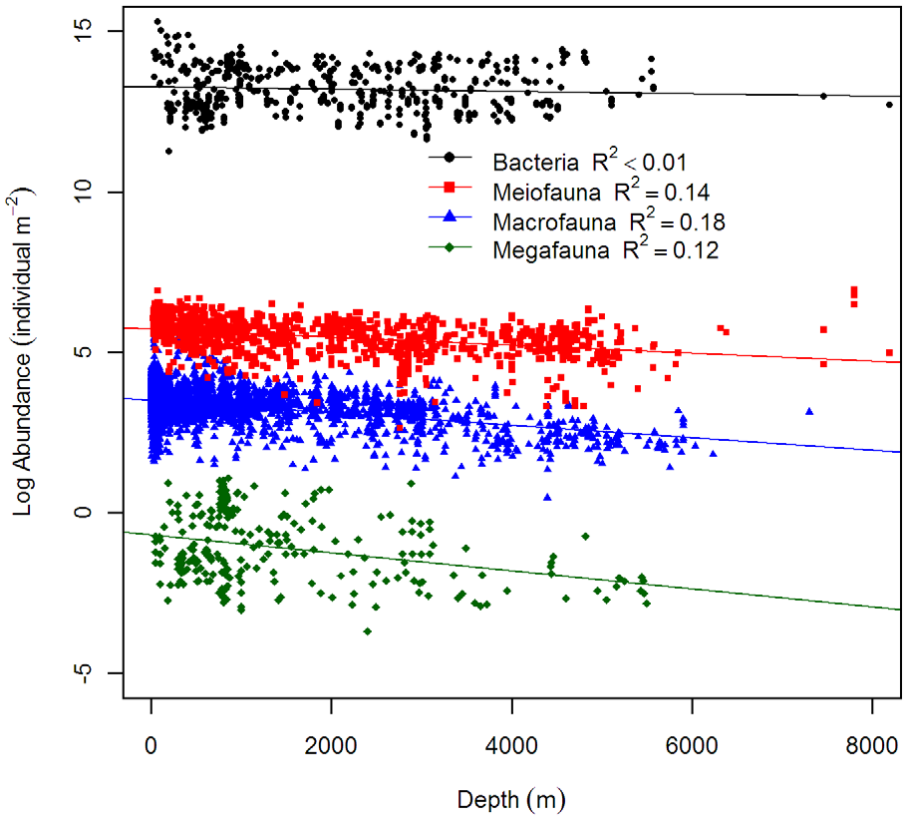
Abundance as a function of depth for bacteria, meiofauna, macrofauna, and megafauna. Abundance was log_10_ transformed and the effects of latitude and longitude were removed by partial regression. Figure legend follows Rex et al. [8] for comparison. References of data source are available in Appendix S1 and File S1. Regression equations and test statistics for each size category are available in Table 2.

**Figure 4:**
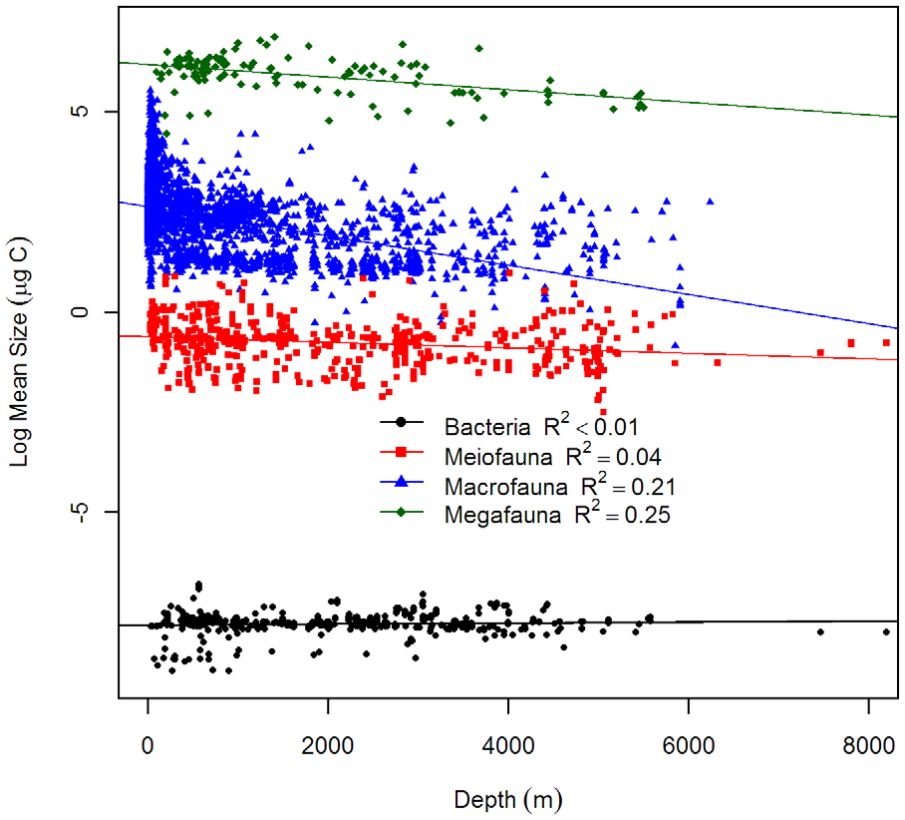
Average body size as a function of depth for bacteria, meiofauna, macrofauna, and megafauna. The average size was calculated by dividing biomass with abundance. The body size was log_10_ transformed and the effects of latitude and longitude were removed by partial regression. Figure legend follows Rex et al. [8] for comparison. References of data source are available in Appendix S1 and File S1. Regression equations and test statistics for each size categories are available in Table 2.

Our results confirmed the conclusions of Rex et al. [8] and suggested significantly negative log-linear relationships of biomass, abundance, and body size for 3 large size classes with depth; however, none of these parameters showed statistically significant depth dependency for bacteria (Table 2). We adapted figure legends from Rex et al. [8] and raised the y-intercepts of their regression equations 3 orders of magnitude (converting the unit from g C m^−2^ to mg C m^−2^) for comparison with our current results. Our regression y-intercepts were slightly lower than the previous synthesis (2.4 vs. 2.5 for bacteria; 2.2 vs. 2.3 for meiofauna; 3.1 vs. 3.2 for macrofauna; 1.8 vs. 2.3 for megafauna.), while the rate of decline biomass with depth was steeper for meiofauna (−2.4×10^−4^ vs. −1.7×10^−4^) and macrofauna (−5.2×10^−4^ vs. −4.5×10^−4^), but more gradual for megafauna (−3.1×10^−4^ vs. −3.9×10^−4^, Table 2). The biomass hierarchy among size groups was similar between the 2 studies: macrofauna dominated the shelves and bacteria and meiofauna dominated the abyssal plains (Figure 2). The only apparent difference was a cross of the regression lines between macrofauna and megafauna at ∼6000 m depth, or a reversal of their biomass hierarchies. The lower y-intercepts and steeper slopes for the meiofauna and macrofauna suggested that the biomass levels were lower in this study than in the previous synthesis. The rate of declining biomass with depth was highest for macrofauna, followed by megafauna and meiofauna. Except for meiofauna, the y-intercept of the abundance-depth regressions were slightly lower in this study (13.3 vs. 14.1 for bacteria; 3.5 vs. 3.6 for macrofauna; −0.7 vs. −0.3 for megafauna.) while the slopes were more gradual (−2×10^−4^ vs. −2.8×10^−4^ for macrofauna; −2.8×10^−4^ vs. −3.7×10^−4^ for megafauna, Table 2). The rate of declining abundance with depth was sharpest for megafauna, followed by macrofauna and meiofauna (Figure 3, Table 2). Average body size for each size class was calculated as biomass divided by abundance. The average sizes of all 3 large groups showed significant depth dependency with the rates of declining mean size with depth being the most rapid for macrofauna, followed by megafauna and meiofauna (Table 2 and Figure 4). The rapid decline in average macrofaunal size was likely overestimated at abyssal depths, because the regression line was apparently higher at shelf depths due to extremely large values (>10 mg C individual^−1^) at high latitude areas.

**Table 2:** Regression analyses of biomass, abundance, and body size against depth for bacteria, meiofauna, macrofauna, and megafauna.

Regression	Equations	N	F
**Log10 Biomass (mg C m^−2^)**			
Bacteria	Y = 2.4−(1.22e−06) X	525	<0.01 n.s.
Meiofauna	Y = 2.18−(2.39e−04) X	689	244.1***
Macrofauna	Y = 3.05−(5.15e−04) X	2552	1885***
Megafauna	Y = 1.81−(3.07e−04) X	282	136.2***
**Log 10 Abundance (individual m^−1^)**			
Bacteria	Y = 13.27−(3.58e−05) X	515	2.82 n.s.
Meiofauna	Y = 5.73−(1.25e−04) X	1148	184.7***
Macrofauna	Y = 3.5−(1.95e−04) X	2734	618.2***
Megafauna	Y = −0.68−(2.82e−04) X	253	32.92***
**Log 10 Body Size(µg C individual^−1^)**			
Bacteria	Y = −7.79+(1.35e−05) X	451	2.28 n.s.
Meiofauna	Y = −0.61−(6.81e−05) X	616	27.6***
Macrofauna	Y = 2.62−(3.63e−04) X	2393	637.3***
Megafauna	Y = 6.17−(1.57e−04) X	136	43.58***

Response variables are log_10_ transformed biomass (mg C m^−2^), abundance (individual m^−1^), and body size (µg C individual^−1^). Predictor is depth (m). Scatter plots of the response variables against predictor and regression lines are given in Figures 2, 3, 4. Abbreviations: N =  number of samples;

***denotes P<0.001; n.s.  =  not significant.

### Random Forests

**Figure 5:**
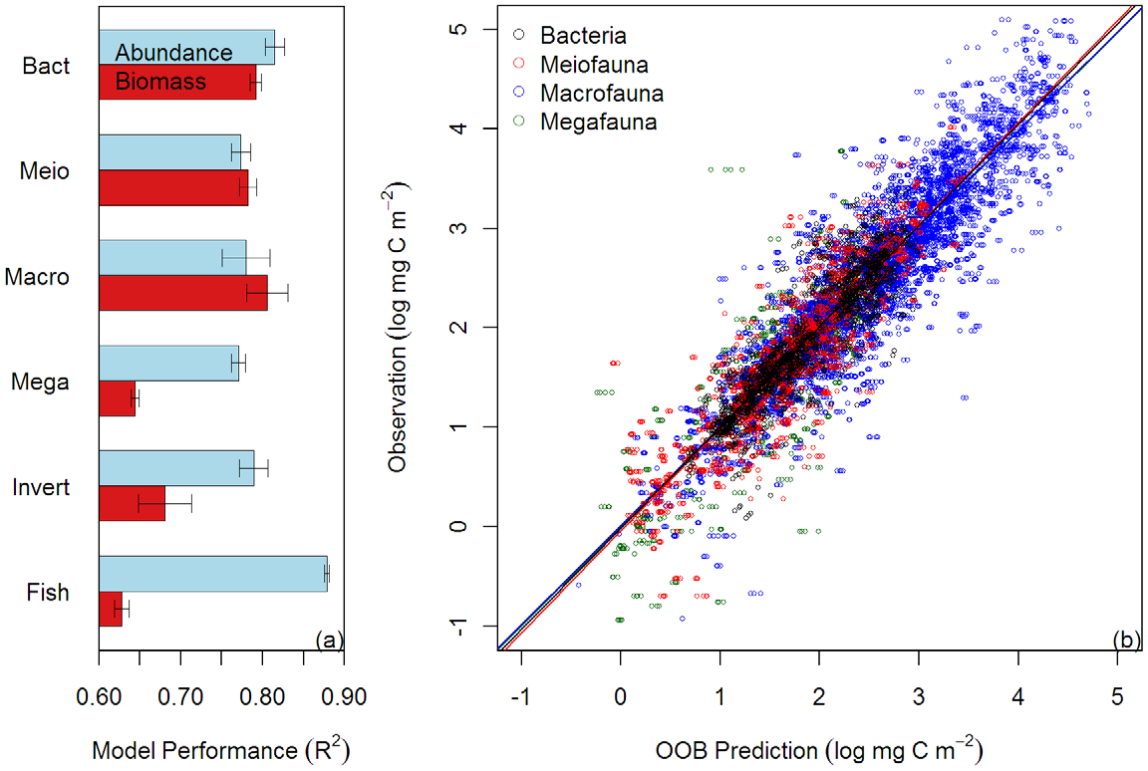
Random Forests (RF) performance on biomass and abundance of each size class. (a) Mean percent variance explained by the RF model ± S.D. (error bar) from 4 RF simulations. Abbreviations: Bact  =  bacteria, Meio  =  meiofauna, Macro  =  macrofauna, Mega  =  megafauna, and invert  =  invertebrates. (b) Observed against OOB predicted biomass from the 4 RF simulations. Color legends indicate 4 major size classes.

On average, RF models explained 78% to 81% of total variance (R^2^) for bacteria, meiofauna, and macrofauna biomass (Figure 5a). Compared to the small size classes, the RF performance was subordinate for megafauna, invertebrates, and fishes, in which the models only explained 63% to 68% of the observed biomass variance. The RF algorithm appears to perform better for abundance with the models explaining 77% to 88% of total variance for each size class. The RF performance among different simulation scenarios was generally stable (S.D ≤1%). The variability was only slightly higher for macrofauna and invertebrates with S.D. between 2% to 3%. A scatter plot between observed and predicted biomass (Figure 5b) suggests that the OOB predictions were in proper scale (regression slopes  = ∼1) with modest deviations from the observations.

**Figure 6:**
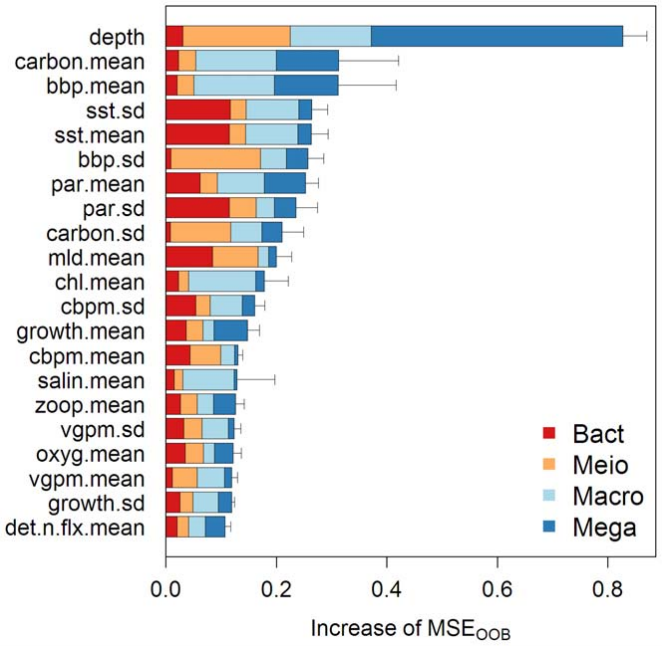
Mean predictor Importance on total seafloor biomass. The predictor importance of major size classes were combined (Figure S3) and mean ± S.D. (error bar) was calculated from 4 RF simulations. The top 20 most important variables are shown in descending order. Increase of mean square error (MSE_OOB_) indicates the contribution to RF prediction accuracy for that variable.

We combined predictor importance from bacteria, meiofauna, macrofauna, and megafauna (Figure S3) to examine the predictor importance on total benthic biomass. This was only done for the biomass datasets because they were converted to a unified currency in mg C per square meter. With the exception of bacteria, depth was ranked highly important for the 3 larger size classes (Figure 6). To our surprise, neither net primary production (vgpm, cbpm) nor flux of detrital organic matter to seafloor (det.c.flx, det.n.flx) was considered the most important for the total benthic biomass. Instead, water depth and the data inputs for the NPP models (carbon, bbp, sst, par, mld, chl) were among the top 10 most important variables. Nonetheless, when the predictor importance was examined for the size classes, NPP models (vgpm, cbpm) had considerable importance for bacterial, meiofaunal, and macrofaunal biomass but appeared less important for megafaunal biomass. Decadal mean and S.D. of the predictors generally ranked in similar orders suggesting high correlation between them; however, it may also suggest that overall levels and seasonal fluctuations of the predictors were both important in predicting the biomass. The predictors associated with water column processes (Table 1) appeared not significant to the total biomass; however, the decadal mean of water column-integrated zooplankton (zoop.mean), total organic matter (int.c.mean, int.n.mean), and detrital organic matter (det.c.mean, det.n.mean), were among the most important predictors for megafaunal standing stocks, especially for abundance (see Figure S3d and Figure S4d). Annual mean salinity (salin.mean) was the only bottom water property ranked within the top 10 most important predictors for the total biomass (Figure 6).

### Patterns of Predicted Biomass

**Figure 7:**
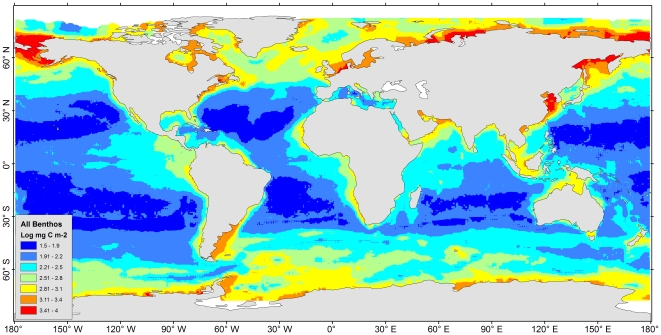
Distribution of seafloor biomass predictions. The total biomass was combined from predictions of bacteria, meiofauna, macrofauna, and megafauna biomass (Figure S5a, b, c, d). Map was smoothed using Inverse Distance Weighting interpolation to 0.1 degree resolution and displayed in logarithm scale (base of 10).

**Figure 8:**
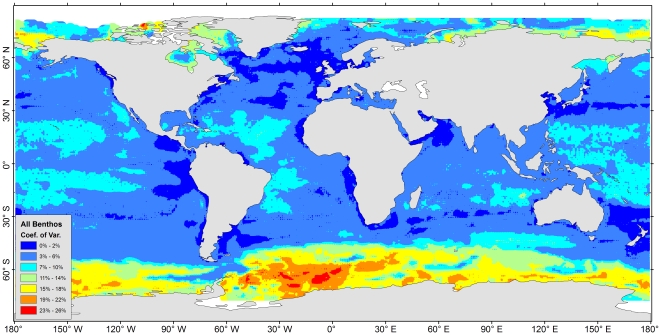
Coefficient of variation (C.V.) for mean seafloor biomass prediction. The C.V. was computed as S.D./mean * 100% from 4 RF simulations. Map was smoothed using Inverse Distance Weighting interpolation to 0.1 degree resolution.

No biomass predictions were given near the northern tip of the Arctic Ocean and part of the Antarctic shores due to a lack of SeaWiFS satellite data as a result of permanent sea ice cover (Figure S2). The predictions of major size classes (Figure S5a, b, c, d) were combined to estimate the total benthic biomass. The maximum biomass of 2.6 to 10 g C per square meter occurred on the shelves of the north frigid zones (e.g. Kara Sea, Siberian Sea, and Chukchi/Bering Sea) and temperate areas (e.g. Yellow sea and North Sea, see Figure 7, red color). These predictions however were lower than the empirical maximum found in the Chukchi/Bering Sea, where the infauna biomass as high as 40 to 100 g C m^−2^ were reported [31]. The discrepancy is probably associated with high prediction uncertainty in the areas (C.V.  = 15% to 22%, Figure 8) or unexplained variability in the models (Figure 5a). The weaker maximum (orange color) between 1.3 to 2.5 g C per square meter occurred on the polar to temperate shelves and subtropical coastal areas (e.g. East/South China Sea, Arabian Sea, and Persian Gulf). The lowest biomass prediction between 30 and 80 mg C per square meter occurred on the abyssal plains of the Pacific, Atlantic, and Indian Ocean; however, relatively higher biomass was predicted on the seafloor of the east side of Pacific and Atlantic basins under the productive equatorial divergence and coastal upwelling areas [32]. For these largest ocean areas, the model outputs were stable among 4 RF simulations with S.D. less than 10% of the mean predictions (Figure 8, light blue to dark blue colors). Any high uncertainties were usually associated with high predicted biomass. The Southern Ocean for example had the highest uncertainty with S.D. between 15% and 26% of the mean (yellow to red class), where most of the uncertainty was derived from the unstable predictions for macrofauna biomass (Figure S6). The S.D. of some Arctic shelves were slightly lower than the Southern Ocean, mostly between 11% and 18% of the mean (green to yellow class, Figure 8). The log_10_ predictied biomass (mg C m^−2^) and abundance (individual m^−2^) for each size class are available in File S2 and File S3, respectively. Global maps showing the mean of abundance prediction and coefficient of variation for each size class are given in Figure S7 and Figure S8, respectively.

**Figure 9:**
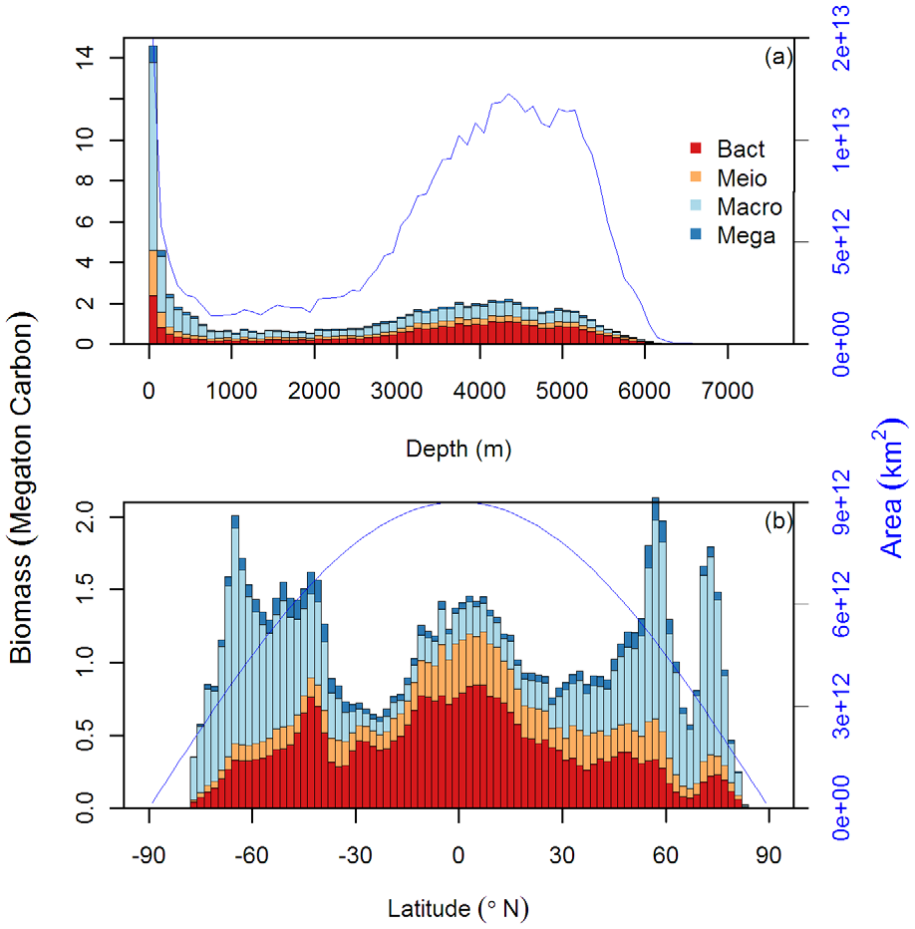
Global zonal integrals of benthic biomass (bars) in unit of megaton carbon based on 100-m bins (a) and 2-latitude-degree bins (b). The blue line shows integrals of seafloor area in unit of square kilometer. Color legends indicate 4 major size classes.

A total of 110.3±48.2 (Mean ± S.D. from 4 RF simulations) megatons of living carbon biomass were estimated based on the global integral of the predicted map cells (Figure 7), in which bacteria, meiofauna, macrofauna, and megafauna contributed 31.4%, 12.9%, 50.7%, and 5% of the global integral, respectively. Previous workers estimated that global POC flux to the seafloor was 3.76 to 3.91 megaton C day^−1^ [25], [33] and carbon burial was about 0.82 megaton C day^−1^ [33]. By dividing the total mass by the flux [34], [35], we estimated that the mean residence time for the seafloor living carbon was 36.6±16 days (mean ±S.D.). Generally, the predictions were highest on the continental shelves, which account for 21.1% of the global integral biomass but cover merely 5.9% of the total seafloor area (≤200 m water depth, Figure 9a). Water depths deeper than 3000 m harbor more then 50% of the global benthic biomass due to their vast area (covering >75% of seafloor). The predictions were also high at high latitudes (> 60°N or S) and the tropical ocean (<23.5°N or S) of the northern and southern hemisphere, in which the biomass contributed 25.4% and 28.8% of the global integral on 13.4% and 40.7% of the ocean area, respectively (Figure 9b). As a rule of thumb, the total biomass of all size classes (except for bacteria) dissipates along the continental margins to the abyssal plains (Figure 2) but this is accompanied by a major shift in size classes in the predictions, with the biomass composition changing from metazoan dominated (meiofauna + macrofauna) for the first couple hundred-meter zonal integrals to bacteria dominated on the abyssal plain (Figure 9a). Along the latitudinal zonal integrals, the biomass composition also shifted from the majority of large-size macrofauna at high latitudes to the small-size meiofauna and bacteria dominated at the tropics (Figure 9b).

**Figure 10:**
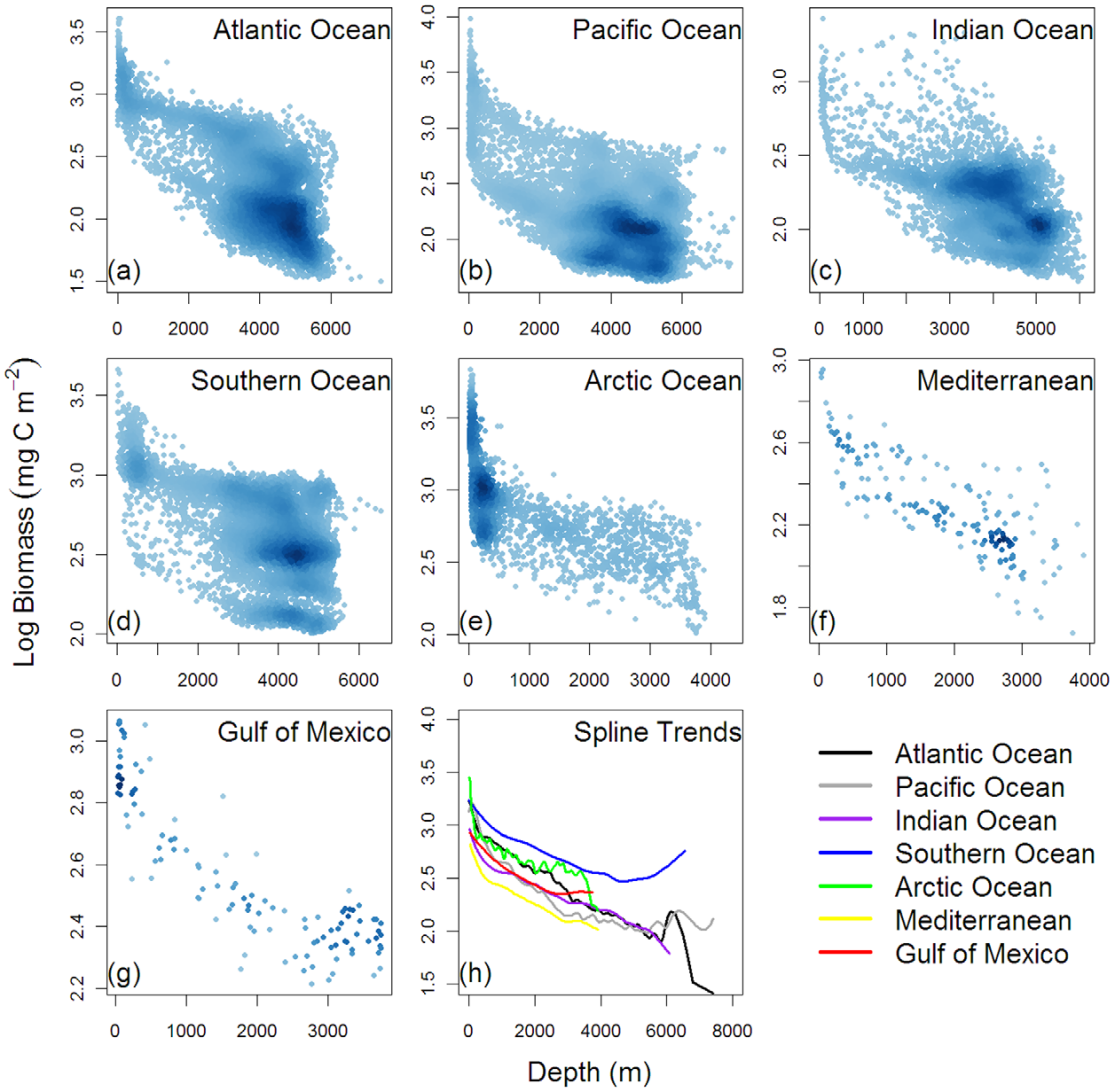
Seafloor biomass predictions against depths for the (a) Atlantic Ocean, (b) Pacific Ocean, (c) Indian Ocean, (d) Southern Ocean, (e) Arctic Ocean, (f) Mediterranean Sea, and (g) Gulf of Mexico. Blue color gradient indicates kernel density estimates. Panel (h) shows the regional predicted trends based on smoothing spline function. Color legend indicates the spline trends for each basin.

Regional variability among the major ocean basin is apparent when predicted biomass was plotted against depth (Figure 10). Generally, the declining trends of biomass with depth were similar but the overall levels differed by basin, with the predictions bounded between the higher end of the Southern Ocean and the lower end of the Mediterranean Sea (Figure 10h). In the Atlantic and Arctic Ocean, high density at bathyal depths near the upper end of the biomass–depth distribution (Figure 10a, e) appeared responsible for elevated biomass levels above the Pacific, Indian Ocean, and Gulf of Mexico (Figure 10). These high values corresponded to the high biomass predictions in the North Atlantic to Arctic Ocean (Figure 7) under the productive subpolar gyre north of the Gulf Stream [32]. The high density at the bottom of the biomass-depth distribution for the Atlantic and Pacific Oceans (Figure 10a, b) illustrates the low predicted biomass on the vast abyssal plains. In the Indian Ocean, the extraordinary high predicted values between ∼1200 to 3000-m water depths (Figure 10c) single out the Oman and Pakistan Margin, where the benthic biomass between 1.3 and 2.5 g C per square meter is as high as continental shelf values (Figure 7, orange color). We believe that the high predictions derive mainly from the monsoon dynamics and seasonal fluctuation of export POC flux [36] rather than the mid-water Oxygen Minimum Zones (OMZ), because resolution of our bottom oxygen data (Table 1) is probably not sufficient to detect OMZ influences. At hadal depths (>6000 m), the biomass predictions were meager in general (<0.2 g C m^−2^, Figure 10a, b); however, relatively high values (0.5∼0.7 g C m^−2^) were predicted near the Kurile-Kamchatka Trench of the Northwest Pacific Basin (Figure 10b) and the South Sandwich Trench near the southern tip of the South America and Antarctic Peninsula (Figure 10d).

## Discussion

### Observed and Predicted Patterns

In this study, classic log-linear declines of seafloor biomass and abundance with depth were demonstrated for meiofauna, macrofauna, and megafauna [7], [8], [9]. These widely recognized patterns have been attributed to the decreasing quantity and quality of sinking phytodetritus with increasing depth and distance from the productive coastal waters and river runoff [7], [37]. Although the selection pressure (food limitation) may be the same, responses differed among the size groups along the depth gradients, showing disparate rates of declining biomass and shifts of biomass hierarchy from macrofauna domination on the shelves and upper slope to meiofauna and bacteria domination on the abyssal plains [8], [34], [35], [38]. Figure 4 suggests that these observed biomass patterns among size groups are governed by the rate of declining average body size rather than by the rate of declining abundance with depth. The decrease of animal size in the deep-sea has been explained by energy constraints and the need to maintain viable density for successful reproduction [8], [39]. Recent evidence from terrestrial environments also suggests a potential link between the animal body size and food quality [40]. It has been suggested that the macrofauna may compete for fresh settled phytodetritus with bacteria [41], [42], [43], [44], while the meiofauna may prefer bacterial carbon over phytodetitus [45]. Hence, the rapid decline of macrofaual average size with depth could be related to the exponential decrease of sinking detrital carbon or the refractory organic matter in the deep-sea sediments. The meiofauna, on the contrary, may be less affected by the deterioration of the food influx and experienced a relatively gradual decline of average size with depth; however, the actual causes of this discrepancy in size-structure remain unclear.

Interestingly, our predicted biomass not only has captured the shifts of dominant size groups with depth but also with latitude (Figure 9), supporting the dominance of macrofaunal biomass [31], [46] and meager importance of bacteria at the high latitudes [47] due, potentially, to strong benthic-pelagic coupling, short food chain, and weaker microbial loop in the overlying water [48], [49]. Other intriguing features from our predictions include the apparent increase of bacterial, meiofaunal, and decrease of macrofaunal biomass integrals from high latitudes toward the tropical oceans (Figure 9b). In fact, the increasing bacterial and meiofaunal integrals were a function of the increasing cell areas toward the equator due to the map projection, which in turn makes the decrease of macrofaunal integrals seemingly even more convincing. This cross-latitude comparison however could be biased by a potential interaction with water depth, because the tropical oceans comprise many deep basins and the high latitudes, such as Chukchi/Bering Sea, have extended shelf areas. We tested this by using partial regression to statistically remove the effect of water depth and longitude. When depth was held constant, macrofaunal biomass could be fitted to a positive parabolic function of latitude (R^2^ = 0.17, P<0.001), supporting the elevated macrofaunal biomass at high latitudes [7].

From a global perspective, the results of regressions (Figures 2, 3, 4) reinforced the weak to no depth-dependency of bacterial standing stocks [8], [50], [51]. Despite immense variation in declining POC flux at depth, the surface sediments supported a remarkably constant bacterial stock spanning only ∼2 orders of magnitude difference worldwide (30 to 2220 mg C m^−2^ and 1.3×10^12^ to 1.9×10^14^ cells m^−2^, 5th to 95th percentile, n = 525); nonetheless, regional and local studies in our database do indicate dependency of bacterial standing stocks with depth or POC flux [10], [52], [53]. The high bacterial stocks at the supposedly depauperate abyssal depths have been attributed to their barophilic adaption [54], [55]. As bacteria are transported with phytoditrital aggregates to the deep sea [56], a large number of the bacteria could be dormant or inactive because of the extreme pressure and frigid temperature [57], [58], while the active microbes are supported by carbon deposition flux [43], viral lysis of the infected prokaryotes [59], extracellular enzymatic activities [60], [61], and benthic metazoan sloppy feeding [44]. It is worth noting that many studies have applied a uniformed conversion factor to estimate the biomass from bacterial numbers, which may be the main reason that no statistical relationship was detected between the bacterial cell size and water depth (Figure 4). Based on direct measurements of the cell volume over a wide range of water depths in the northern Gulf of Mexico, Deming and Carpenter [52] concluded that the greater ocean depths generally harbored smaller bacterial cells despite the abundance remaining constant. That is, the constancy of bacterial biomass with depth that we observed here could be an artifact because the cell volumes were not measured directly at all depths. To our surprise, even though no depth-dependency was evident for the bacterial standing stocks, the RF algorithm performed well in predicting the bacterial biomass (R^2^ = 79±0.6%) and abundance (R^2^ = 81±1.2%, mean±S.D, n = 4). High predictor importance of sea surface temperature (sst), irradiance (par), mixed layer depth (mld), and carbon-based primary production model (cbpm) support the idea that the sedimentary bacterial biomass may be imported in the form of sinking particles [54], [56]. The high bacterial biomass predictions on the abyssal plains of semi-enclosed basins, such as the Gulf of Mexico, Arabian Sea, and East Mediterranean (Figure S5a), supported potential lateral advection of detritus from the margins due to relatively large area of shelves and margins compared to basin volume [52].

### Anomalies not explained by Random Forests

Although multiple predictors were obtained to cover as many aspects and processes that could affect the distribution of benthic standing stocks, around 19% to 36% of observed variances are still unexplainable in the current RF models. Some important predictors, such as sediment grain size [62], organic composition [63], bioturbation [64], [65], and community oxygen demand [66], [67], were not included due to sparse data availability; others such as oxygen minimums [68], [69] or abrupt changes in thermal dynamic regimes [70], could also be left undetected due to the coarse resolution in available hydrographic data. Nevertheless, the largest unexplained variability was probably derived from our non-contemporaneous predictors that do not account for the seasonal or inter-annual changes of benthic standing stocks as a result of climate-induced variations on productivity and export POC flux [71], [72]. The seafloor organisms depend on diverse sources of energy [73], including large food falls [74], hydrocarbons from cold seeps and hydrothermal vents [75], [76], lateral resource advection from continental margins [77], accumulation of organic matter in submarine canyons [78] and trenches [79], and rapid energy transfers on seamounts [80]. In addition, benthic foraminifera, sometimes accounting for more than 50% of eukaryote biomass [81], are not included in our datasets. These anomalies are not in the scope of this analysis and should be estimated separately elsewhere in a global context. For example, at the head of the New Zealand's Kaikoura Canyon (data not in the database), the extremely high macrofauna and megafauna biomass (89 g C m^−2^) was about 100-fold more than our total biomass prediction (0.94 g C m^−2^) [82]. Within the datasets, extraordinary high “total biomass” was also reported at the head of the Mississippi Submarine Canyon [35] due to dominance of a “carpet of worms” [83]. The observed biomass was still more than 4-fold higher than our prediction. This is partially because the Gulf of Mexico basin had very high background bacterial biomass [52]. When the bacteria component is removed, the prediction still under estimates the observed biomass by about 50%. Hence, the total living carbon prediction in this study (Figure 7) should be considered as a conservative estimate for the soft bottom communities solely reling on sinking phytodetritus, with the anomalies causing the observed biomass to deviate from this baseline (Figure 5b).

### Predictor Importance

We tested the RF algorithm using only the primary productivity predictors (decadal mean and S.D. of chl, sst, par, bbp, mld, growth, carbon, vgpm, and cbpm) and depth (Table 1). We found that the reduced models only experienced modest deterioration in performance (R^2^_reduced_  = 75%–80.3% for biomass of 3 small size classes and R^2^_reduced_  = 63% for megafauna biomass; R^2^_reduced_  = 76.3%–80.6% for abundance of 4 major size classes), suggesting that these productivity/depth predictors alone can explain much of the observed stock variances. It is also evident that these satellite-based ocean color parameters and depth are among the most important predictors when the full RF models were constructed (Figure 6). Their importance was even greater than the model estimates of export phytodetritus flux (det.c.flx & det.n.flx, Table 1) that have been considered important for benthic communities [43], [56], [84], [85]. One possibility is that not all export flux is utilized by the benthos [35] and the combination of productivity/depth predictors simply explain the stock variances better; however, the spurious correlations between these predictors could also make them all rank highly important. Strobl et al. [86] recommended “Conditional Permutation” while calculating the variable importance to reduce the effect of spurious correlations. We did not attempt this analysis because our focus was on prediction rather than pinpointing the exact contribution of each predictor.

### Conclusions

The fate of sinking phytodetritus flux to the ocean floor and the energy transfer to the benthos is a complex biogeochemical process. The combination of mechanistic primary productivity models [23], [24] and empirical relationship of export POC flux at depth [87] may not properly reflect the actual benthic food influx and consumption. In this study, we demonstrated that the combination of multivariate predictors and machine-learning algorithm was superior to conventional regression models using only water depth or export POC flux to predict benthic standing stocks [8], [88]. Conceptually, the RF predicted biomass presented here (Figure 7) can be seen as non-linear transformation of the surface primary production through a sophisticated decision network and is thus potentially a more realistic reflection of benthic food supply or utilization. Benthic biomass is essential to understand the dynamic processes of global carbon cycling [89] and productivity-diversity relationship in the deep sea [1], [2]. Predictive mapping of this kind can fill the gaps where critical biomass information is lacking, since a true ‘census’ of global living carbon is expensive and practically impossible. Accurate prediction of benthic biomass can facilitate Ecosystem Based Management (EBM) on socioeconomically important species [90]. It is also extremely useful for generating and testing large scale hypotheses (e.g. latitudinal and cross-basin comparison) and planning shipboard surveys. Moreover, the reduced RF models mentioned above can be used to perform fine-scale predictions with high resolution ocean color images (5 arc minute grids) and the global relief model (1 arc minute grids, Table 1), and potentially reveal more heterogeneous biomass patterns at local scale than the current coarse analysis framework. The ocean color/depth predictors also make it possible to do contemporaneous modeling with recent sampling (SeaWiFS data are only available since 1997) or data collected in the future. This study presents an initial framework for archiving the seafloor standing stock data. More training datasets from diverse environments matched in space and time are urgently needed to improve the model performance and prediction accuracy, and perhaps, in due course, the seafloor standing stocks can be now-casted using the current ocean climate or even forecasted under the future climate scenarios [91].

## Supporting Information

File S1 Google Earth file for the “CoML fresh biomass database”.
(KML)

File S2 Global seafloor biomass predictions. Predicted biomass (mg C m^-2^) is in global 1×1 degree grids. Data fields include latitude, longitude, depth, and biomass of each size class. The biomass data are in logarithm scale (base 10).
(CSV)

File S3 Global seafloor abundance predictions. Predicted abundance (individual m-^2^) is in global 1×1 degree grids. Data fields include latitude, longitude, depth, and abundance of each size class. The abundance data are in logarithm scale (base 10).
(CSV)

Appendix S1 The complete list of references for the “CoML Fresh Biomass Database”.
(DOC)

Figure S1 Environmental predictors for Random Forest models. Data were logarithm transformed (base 10) and scaled to between 0 (minimum value) and 1 (maximum value). Detail description of the variable is given in Table 1. Abbreviations: mean  =  decadal or annual mean; sd  =  decadal or seasonal standard deviation.
(TIFF)

Figure S2 Temporal coverage of primary productivity predictors between years of 1998 and 2007. Color ramp shows the sample size from 0 to 120 months of measurements. Detail description of the variable is given in Table 1. Abbreviations: n  =  sample size.
(TIFF)

Figure S3 Mean predictor Importance for biomass of (a) bacteria, (b) meiofauna, (c) macrofauna, and (d) megafauna. The mean ± S.D. (error bar) were calculated from 4 RF simulations. The top 20 most important variables are shown in descending order. Increase of mean square error (IncMSE) indicates the contribution to RF prediction accuracy for that variable. Detail description of the variable is given in Table 1. Abbreviations: mean  =  decadal or annual mean; sd  =  decadal or seasonal standard deviation.
(TIF)

Figure S4 Mean predictor Importance for abundance of (a) bacteria, (b) meiofauna, (c) macrofauna, and (d) megafauna. The mean ± S.D. (error bar) were calculated from 4 RF simulations. The top 20 most important variables are shown in descending order. Increase of mean square error (IncMSE) indicates the contribution to RF prediction accuracy for that variable. Detail description of the variable is given in Table 1. Abbreviations: mean  =  decadal or annual mean; sd  =  decadal or seasonal standard deviation.
(TIF)

Figure S5 Distribution of mean biomass predictions for (a) bacteria, (b) meiofauna, (c) macrofauna, (d) megafauna, (e) invertebrates, and (f) fishes. The mean biomass was computed from 4 RF simulations. Predictions were smoothed by Inverse Distance Weighting interpolation to 0.1 degree resolution and displayed in logarithm scale (base of 10).
(TIF)

Figure S6 Coefficient of variation (C.V.) for mean biomass predictions of each size class. The C.V. was computed as S.D./mean * 100% from 4 RF simulations. The abbreviations are: bact  =  bacteria, meio  =  meiofauna, macro  =  macrofauna, mega  =  megafauna, inv  =  invertebrates, fis  =  fishes.
(TIFF)

Figure S7 Distribution of mean abundance predictions for (a) bacteria, (b) meiofauna, (c) macrofauna, (d) megafauna, (e) invertebrates, and (f) fishes. The mean abundance was computed from 4 RF simulations. Predictions were smoothed by Inverse Distance Weighting interpolation to 0.1 degree resolution and displayed in logarithm scale (base of 10).
(TIF)

Figure S8 Coefficient of variation (C.V.) for mean abundance predictions of each size class. The C.V. was computed as S.D./mean * 100% from 4 RF simulations. The abbreviations are: bact  =  bacteria, meio  =  meiofauna, macro  =  macrofauna, mega  =  megafauna, inv  =  invertebrates, fis  =  fishes.
(TIFF)
