# Supplementary material for: Global Patterns and Predictions of Seafloor Biomass Using Random Forests
Source: PLoS One. 2010 Dec 30;5(12):e15323. doi: 10.1371/journal.pone.0015323 (PMC3012679; doi:10.1371/journal.pone.0015323)
Supplement: Appendix S1 — The complete list of references for the “CoML Fresh Biomass Database”. (DOC) [file pone.0015323.s004.doc]

| **Size Class** | **Dataset** | **Ocean/Sea** | **References** |
| --- | --- | --- | --- |
|  |  |  |  |
| **Bacteria** | Biomass | Arabian Sea |  |
|  |  | Arctic Ocean |  |
|  |  | Atlantic Ocean |  |
|  |  | Black Sea |  |
|  |  | Caribbean Sea |  |
|  |  | Gulf of Mexico |  |
|  |  | Mediterranean |  |
|  |  | Pacific Ocean |  |
|  |  |  |  |
|  | Abundance | Arabian Sea |  |
|  |  | Arctic Ocean |  |
|  |  | Atlantic Ocean |  |
|  |  | Black Sea |  |
|  |  | Caribbean Sea |  |
|  |  | Gulf of Mexico |  |
|  |  | Mediterranean |  |
|  |  | Pacific Ocean |  |
|  |  |  |  |
|  |  |  |  |
| **Meiofauna** | Biomass | Arctic Ocean |  |
|  |  | Atlantic Ocean |  |
|  |  | Caribbean Sea |  |
|  |  | Gulf of Mexico |  |
|  |  | Indian Ocean |  |
|  |  | Mediterranean Sea |  |
|  |  | Pacific Ocean |  |
|  |  | Southern Ocean |  |
|  |  |  |  |
|  | Abundance | Arctic Ocean |  |
|  |  | Atlantic Ocean |  |
|  |  | Caribbean Sea |  |
|  |  | Gulf of Mexico |  |
|  |  | Indian Ocean |  |
|  |  | Mediterranean Sea |  |
|  |  | Pacific Ocean |  |
|  |  | Red Sea |  |
|  |  | Southern Ocean |  |
|  |  |  |  |
|  |  |  |  |
| **Macrofauna** | Biomass | Arctic Ocean |  |
|  |  | Atlantic Ocean |  |
|  |  | Baltic Sea |  |
|  |  | Bohai/East China Sea |  |
|  |  | Caribbean Sea |  |
|  |  | Gulf of Mexico |  |
|  |  | Indian Ocean |  |
|  |  | Pacific Ocean |  |
|  |  | Southern Ocean |  |
|  |  |  |  |
|  | Abundance | Arctic Ocean |  |
|  |  | Atlantic Ocean |  |
|  |  | Baltic Sea |  |
|  |  | Bohai/East China Sea |  |
|  |  | Caribbean Sea |  |
|  |  | Gulf of Mexico |  |
|  |  | Indian Ocean |  |
|  |  | Mediterranean Sea |  |
|  |  | Pacific Ocean |  |
|  |  | Southern Ocean |  |
|  |  |  |  |
|  |  |  |  |
| **Megafauna** | Biomass | Atlantic Ocean |  |
|  |  | Caribbean Sea |  |
|  |  | Gulf of Mexico |  |
|  |  | Mediterranean |  |
|  |  | Pacific Ocean |  |
|  |  |  |  |
|  | Abundance | Atlantic Ocean |  |
|  |  | Caribbean Sea |  |
|  |  | Gulf of Mexico |  |
|  |  | Mediterranean |  |
|  |  | Pacific Ocean |  |
|  |  |  |  |
|  |  |  |  |
| **Invertebrates** | Biomass | Arctic Ocean |  |
|  |  | Atlantic Ocean |  |
|  |  | Gulf of Mexico |  |
|  |  | Mediterranean |  |
|  |  | Pacific Ocean |  |
|  |  |  |  |
|  | Abundance | Arctic Ocean |  |
|  |  | Atlantic Ocean |  |
|  |  | Gulf of Mexico |  |
|  |  | Mediterranean |  |
|  |  | Pacific Ocean |  |
|  |  |  |  |
|  |  |  |  |
| **Fishes** | Biomass | Atlantic Ocean |  |
|  |  | Gulf of Mexico |  |
|  |  | Mediterranean |  |
|  |  | Pacific Ocean |  |
|  |  |  |  |
|  | Abundance | Atlantic Ocean |  |
|  |  | Gulf of Mexico |  |
|  |  | Mediterranean |  |
|  |  | Pacific Ocean |  |
|  |  |  |  |
|  |  |  |  |
| **Nematodes** | Biomass & Abundance | Atlantic Ocean |  |
|  |  | Gulf of Mexico |  |
|  |  | Indian Ocean |  |
|  |  | Mediterranean |  |
|  |  |  |  |
|  |  |  |  |
| **Pelagic Decapods** | Biomass & Abundance | Mid-Atlantic Ridge |  |
|  |  |  |  |
