# Supplementary figures and images for: Global Patterns and Predictions of Seafloor Biomass Using Random Forests
Source: PLoS One. 2010 Dec 30;5(12):e15323. doi: 10.1371/journal.pone.0015323 (PMC3012679; doi:10.1371/journal.pone.0015323)

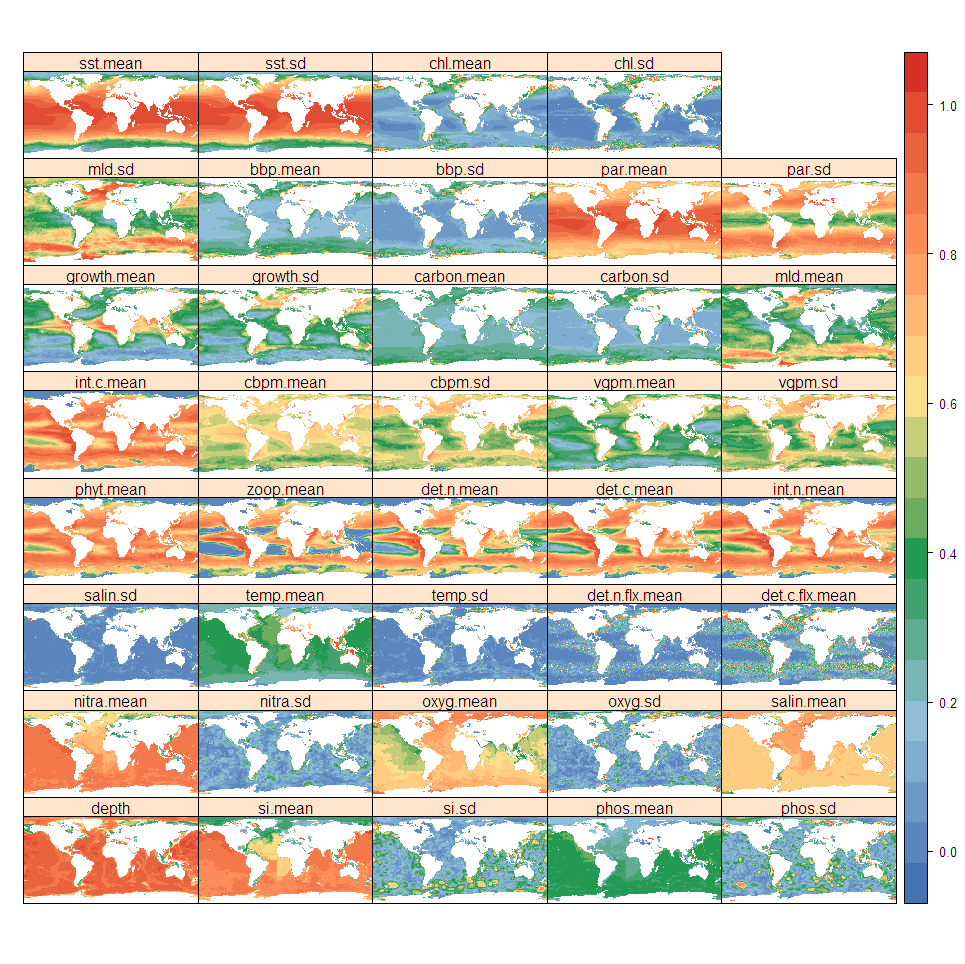

Supplement: Figure S1 — Environmental predictors for Random Forest models. Data were logarithm transformed (base 10) and scaled to between 0 (minimum value) and 1 (maximum value). Detail description of the variable is given in Table 1. Abbreviations: mean = decadal or annual mean; sd = decadal or seasonal standard deviation. (TIFF) [file pone.0015323.s005.tif]

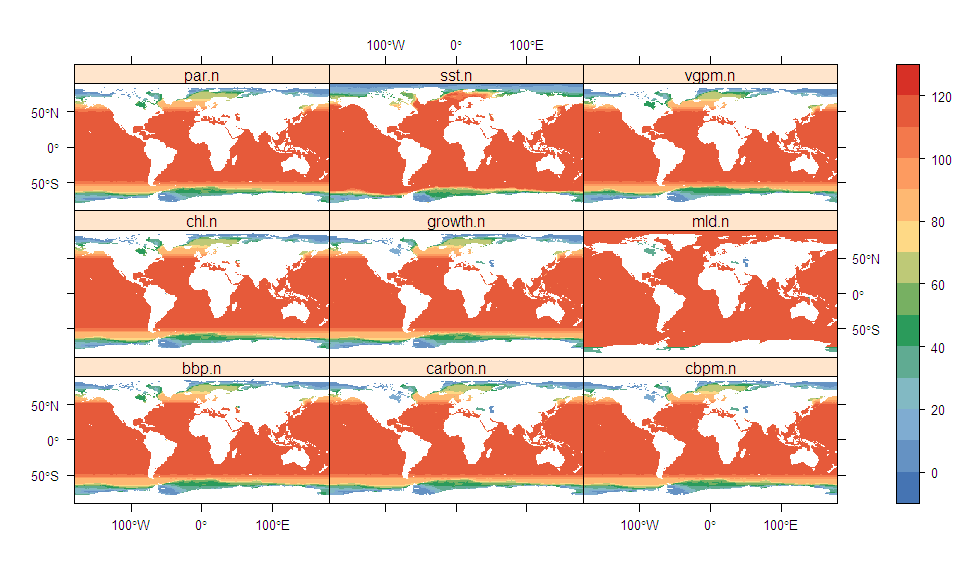

Supplement: Figure S2 — Temporal coverage of primary productivity predictors between years of 1998 and 2007. Color ramp shows the sample size from 0 to 120 months of measurements. Detail description of the variable is given in Table 1. Abbreviations: n = sample size. (TIFF) [file pone.0015323.s006.tif]

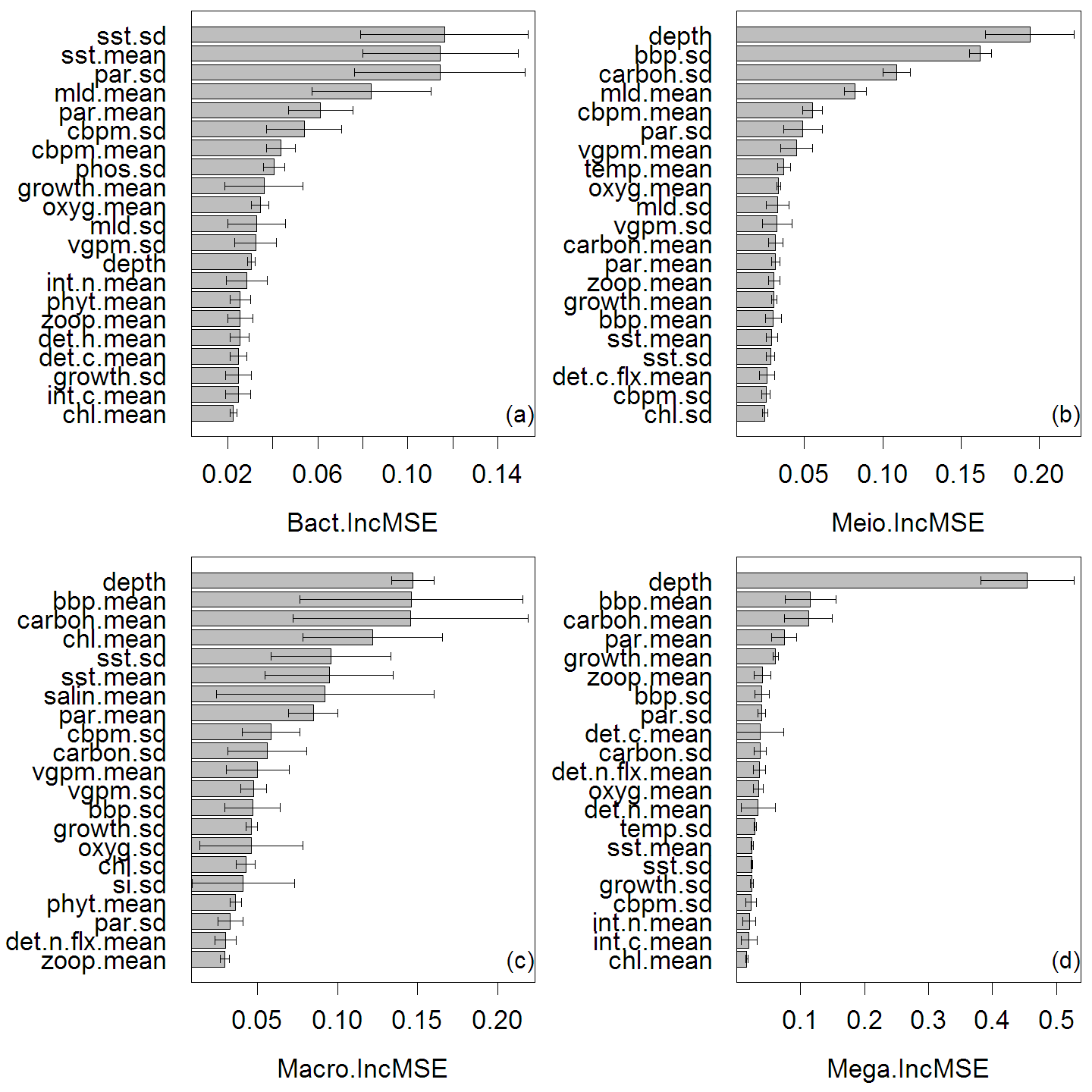

Supplement: Figure S3 — Mean predictor Importance for biomass of (a) bacteria, (b) meiofauna, (c) macrofauna, and (d) megafauna. The mean ± S.D. (error bar) were calculated from 4 RF simulations. The top 20 most important variables are shown in descending order. Increase of mean square error (IncMSE) indicates the contribution to RF prediction accuracy for that variable. Detail description of the variable is given in Table 1. Abbreviations: mean = decadal or annual mean; sd = decadal or seasonal standard deviation. (TIF) [file pone.0015323.s007.tif]

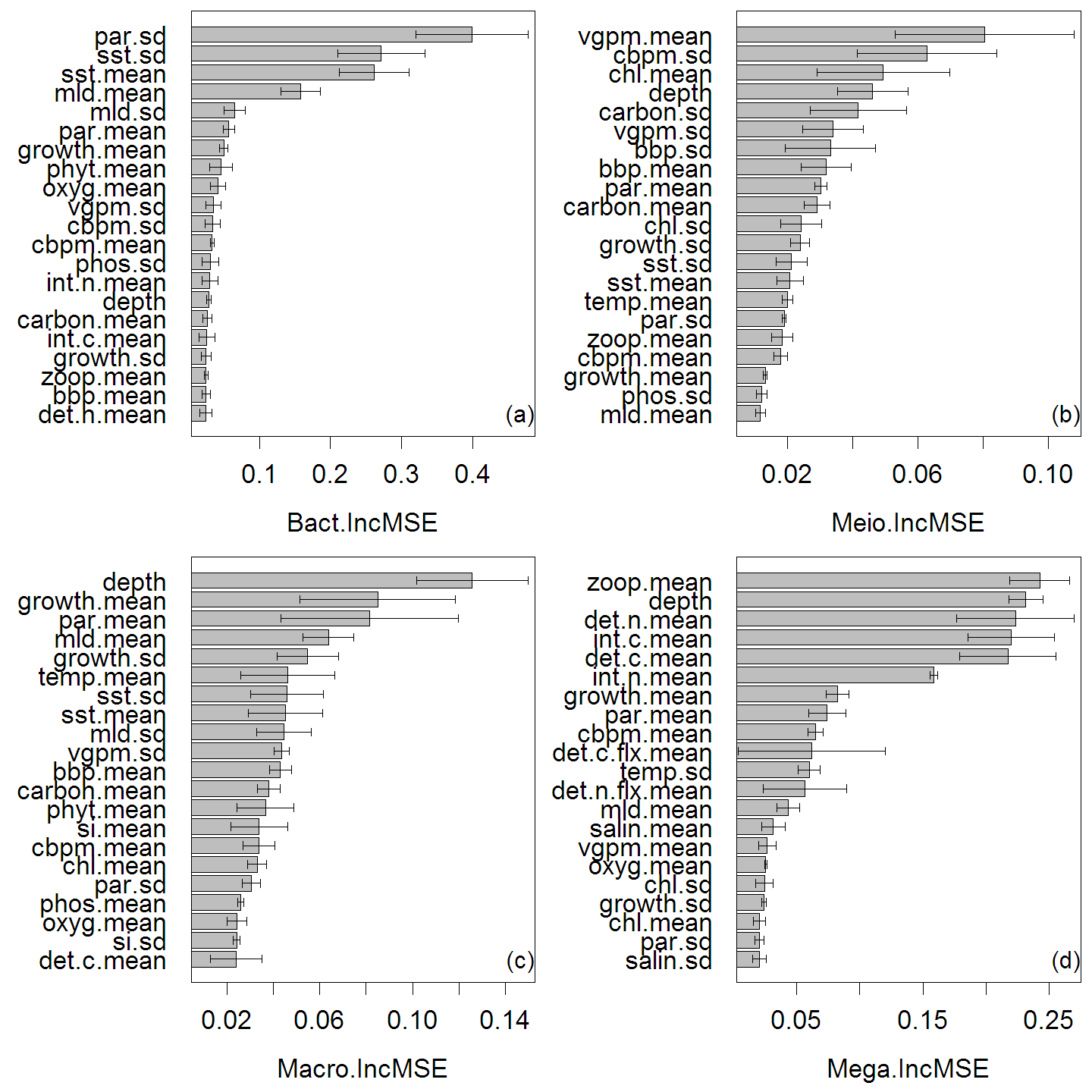

Supplement: Figure S4 — Mean predictor Importance for abundance of (a) bacteria, (b) meiofauna, (c) macrofauna, and (d) megafauna. The mean ± S.D. (error bar) were calculated from 4 RF simulations. The top 20 most important variables are shown in descending order. Increase of mean square error (IncMSE) indicates the contribution to RF prediction accuracy for that variable. Detail description of the variable is given in Table 1. Abbreviations: mean = decadal or annual mean; sd = decadal or seasonal standard deviation. (TIF) [file pone.0015323.s008.tif]

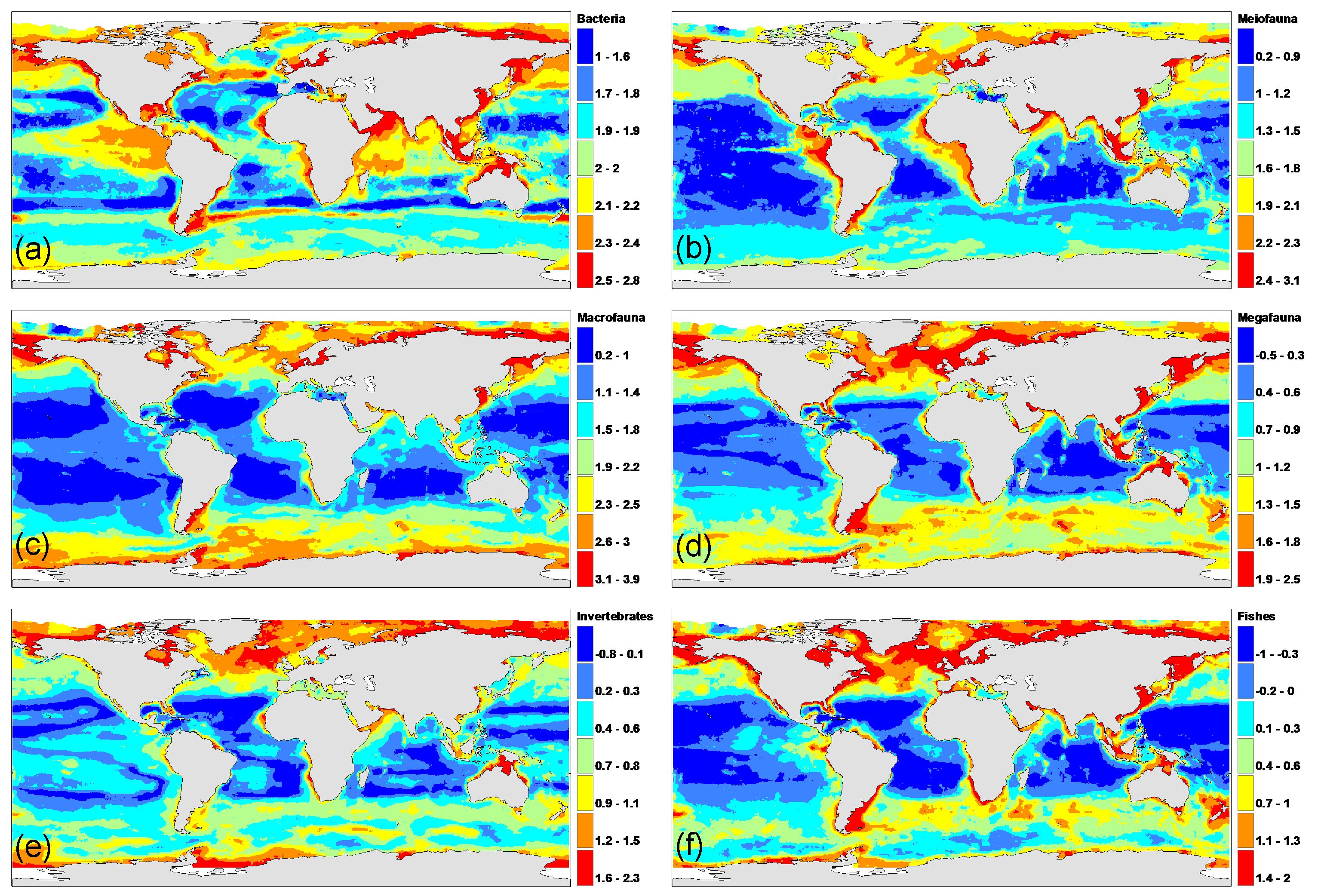

Supplement: Figure S5 — Distribution of mean biomass predictions for (a) bacteria, (b) meiofauna, (c) macrofauna, (d) megafauna, (e) invertebrates, and (f) fishes. The mean biomass was computed from 4 RF simulations. Predictions were smoothed by Inverse Distance Weighting interpolation to 0.1 degree resolution and displayed in logarithm scale (base of 10). (TIF) [file pone.0015323.s009.tif]

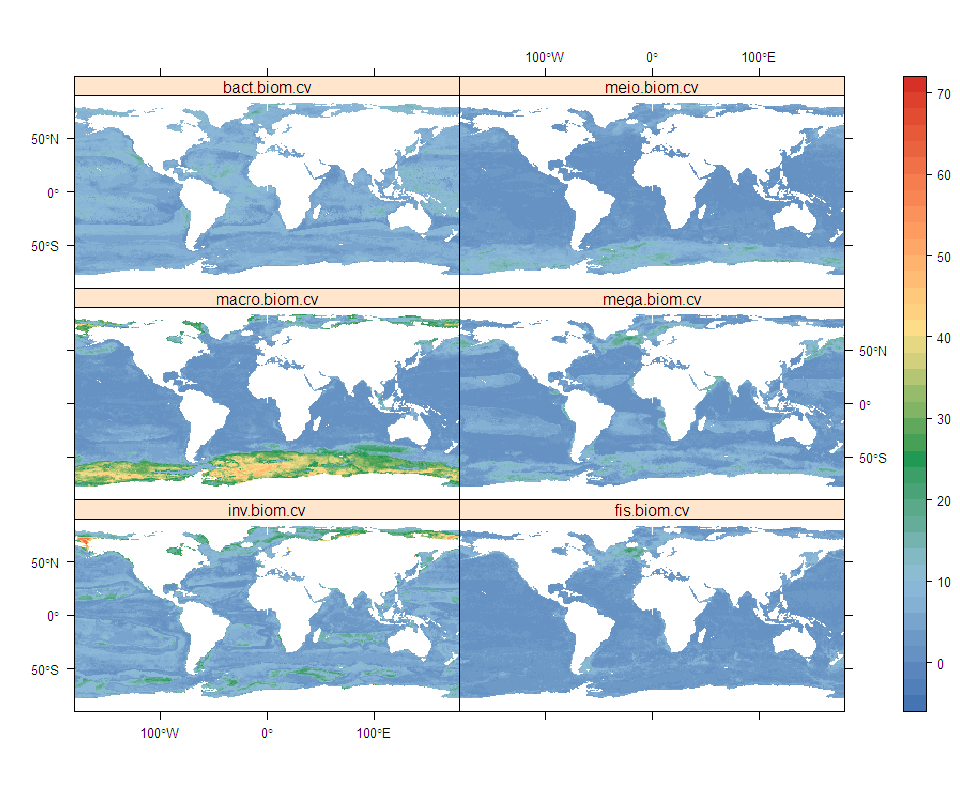

Supplement: Figure S6 — Coefficient of variation (C.V.) for mean biomass predictions of each size class. The C.V. was computed as S.D./mean * 100% from 4 RF simulations. The abbreviations are: bact = bacteria, meio = meiofauna, macro = macrofauna, mega = megafauna, inv = invertebrates, fis = fishes. (TIFF) [file pone.0015323.s010.tif]

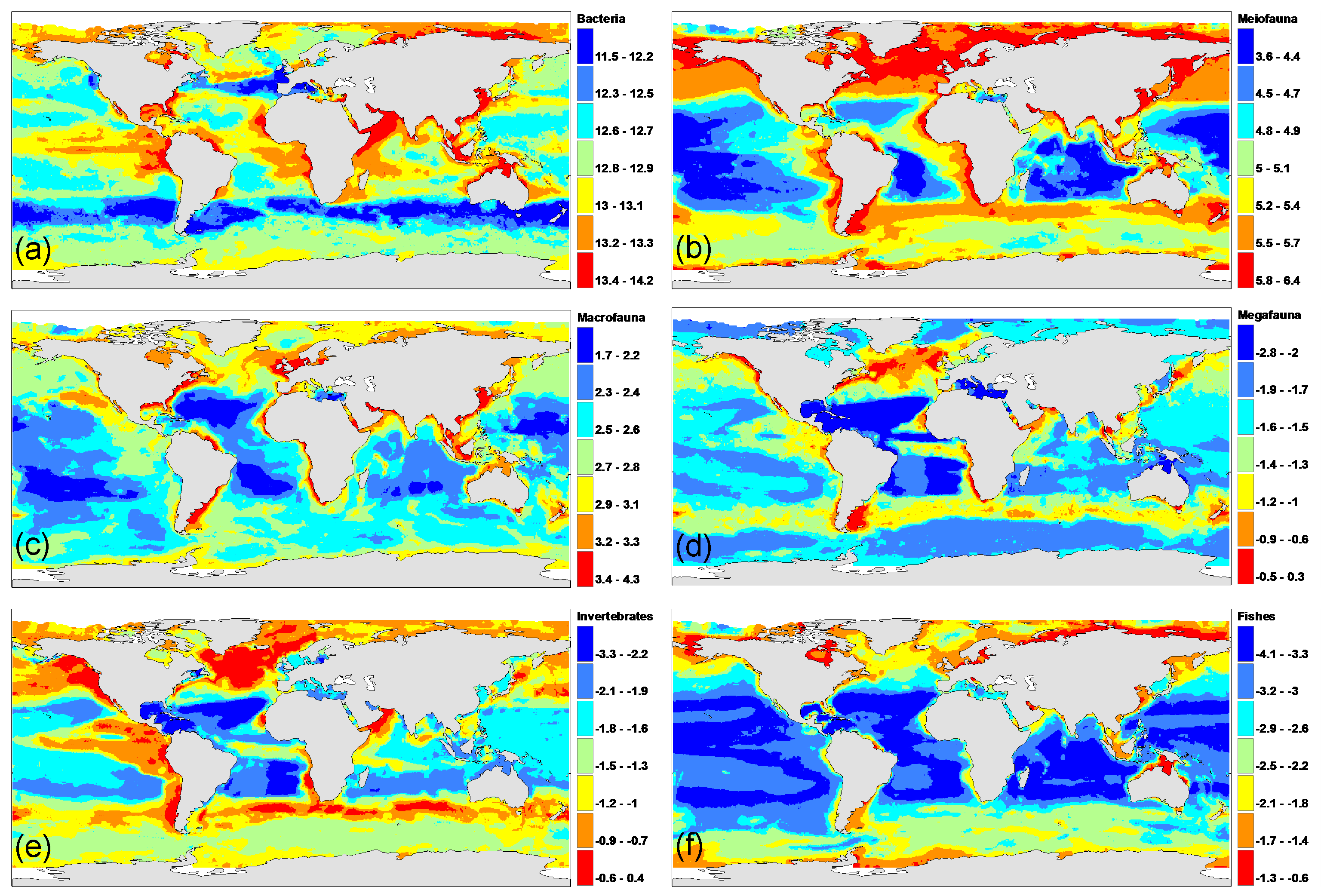

Supplement: Figure S7 — Distribution of mean abundance predictions for (a) bacteria, (b) meiofauna, (c) macrofauna, (d) megafauna, (e) invertebrates, and (f) fishes. The mean abundance was computed from 4 RF simulations. Predictions were smoothed by Inverse Distance Weighting interpolation to 0.1 degree resolution and displayed in logarithm scale (base of 10). (TIF) [file pone.0015323.s011.tif]

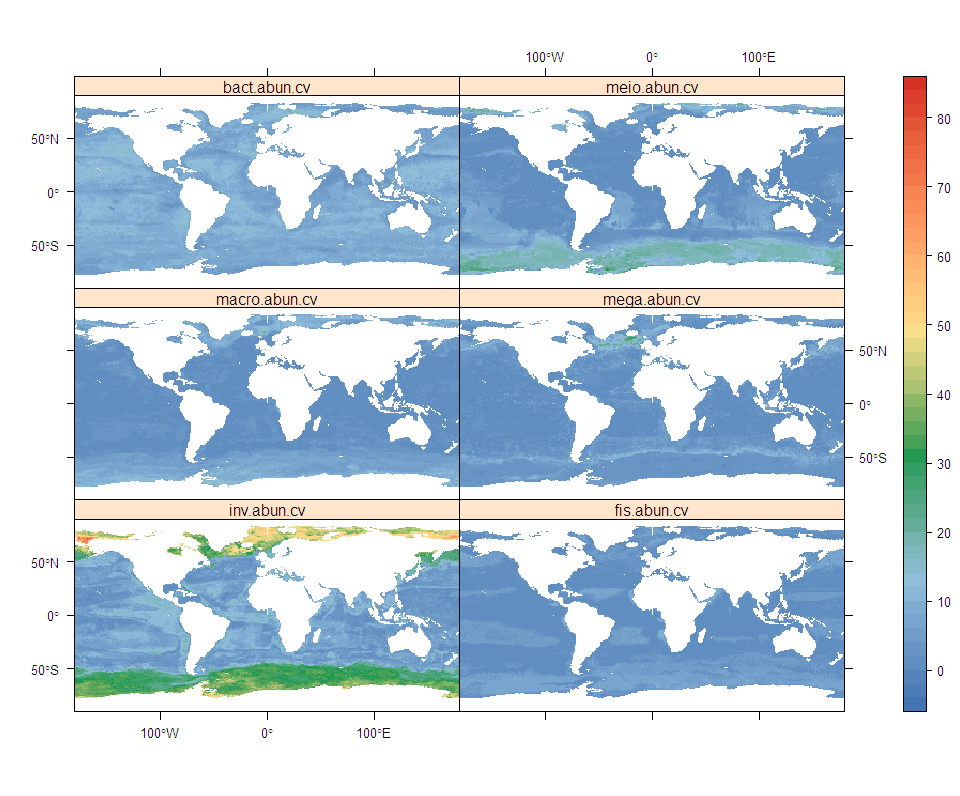

Supplement: Figure S8 — Coefficient of variation (C.V.) for mean abundance predictions of each size class. The C.V. was computed as S.D./mean * 100% from 4 RF simulations. The abbreviations are: bact = bacteria, meio = meiofauna, macro = macrofauna, mega = megafauna, inv = invertebrates, fis = fishes. (TIFF) [file pone.0015323.s012.tif]
